# Climate change and vulnerability discourse by students at a South African university

**DOI:** 10.4102/jamba.v10i1.476

**Published:** 2018-05-07

**Authors:** Shingirai S. Mugambiwa, Obey Dzomonda

**Affiliations:** 1 Department of Sociology and Anthropology, University of Limpopo, South Africa; 2 Department of Business Management, University of Limpopo, South Africa

## Abstract

Climate change is expected to pose grave consequences to communities around the world. It is predicted that many people, mostly in the developing world, will experience shortages of water and food as well as numerous health-related effects because of climate change. Therefore, rigorous global action is needed to enable developing countries to adapt to the effects of climate change. Universities play a pivotal role in addressing these issues and their impacts through research and technological innovations. Hence, assessing the extent to which university students understand climate change and its impacts displays the extent of hope in mitigating future changes in climatic conditions. This article assesses the knowledge and understanding of climate change and its impacts by students at an institution of higher learning in South Africa. This study utilised a quantitative approach and a descriptive design. The convenience method was used to obtain participants for the study. Self-administered questionnaires were utilised in a survey to collect data from the participants. A sample of 90 university students participated in the survey. Data analysis included descriptive statistics and *T*-tests. Reliability was measured using the Cronbach’s alpha. The study discovered that university students have low knowledge and understanding of climate change. As a result, the study concluded that if students could be well-informed about climate change issues, they could positively contribute to the development of their communities by crafting smart climate change mitigation and adaptation skills.

## Introduction

Climate change and its impacts have grave consequences on communities and people’s livelihoods. Weber (2010) defines climate change as systematic changes in average weather conditions over time. The changes are difficult to observe and could not be statistically measured; hence, it is difficult for sceptics to believe climate change is happening. The knowledge and awareness of climate change by university students is of paramount importance as it equips them with skills to deal with its future impacts. El Zoghbi and El Ansari (2014) assert that young people will experience the impact of climate challenges in their future families and communities. Hence, it will be imperative for them to deal with climate impacts such as extreme weather events and rising temperatures as they are expected to be extreme and frequent. The implications of climate change are likely to outspread beyond physical impacts to other aspects such as youth’s emotional health, social inclusion, academic and professional functioning of life. This addresses the limitations of a definition of climate change by Weber and Stern (2011) who defined climate change as a set of physical phenomena and public policy issue which is also referred to as global warming. As a result, it is apparent that the effects of climate change should not be limited to physical phenomena and policy issues; rather, it should be related to a plethora of aspects. For instance, Mugambiwa and Tirivangasi (2017) are of the view that climate change is likely to reduce the role of agriculture to the gross domestic product (GDP) and pose a serious threat to national food security and the economy. Consequently, it is apparent that the effects of climate change can be classified into many distinct categories and as a result the phenomenon should be broadly defined.

The quality of life of university students can be influenced by the type of information they receive on climate change. To explain, the information, in whatever form it comes, shapes their sense of efficacy and agency to inspire changes within their communities. University students are ardent technology users with potential to make important contributions to climate change mitigation (El Zoghbi & El Ansari 2014). Although the impacts of climate change are more severe in the developing world, it is imperative for university students in developing countries to have great awareness and knowledge of the impacts of climate change such that they will be able to devise ways to mitigate climate change. Students are the future leaders. Hence, having a generation which understands the effects of climate change enables them to influence policy. Students play an important role in educating the citizenry on climate change in their various localities. However, awareness and knowledge of climate change vary in many respects. O’Connor et al. (2002) assert that awareness and knowledge of climate change are higher amongst men, graduates and middle-aged people. In both the UK and US studies, men were found to be more aware of the impacts and causes of climate change, whereas a majority of women somehow showed less knowledge about the causes of climate change (Bibbings 2004). Also, DEFRA (2003) asserts that graduates are more likely to have heard of climate change as compared to non-graduates. This implies that university students or those who have a higher level of education are more likely to have an impact on climate change (Eurobarometer 2001). This was supported by Ayanlade (2016) who found that a large number of graduates from Nigerian universities have knowledge about climate change and they developed awareness of climate change through different channels.

Hargreaves, Lewis and Speers (2003) posit that people with a formal science qualification have a higher understanding of the process through which climate change works. This implies that not all students in universities have knowledge about how climate change works, that is, the causes, impacts as well as mitigation measures. It has also been discovered that awareness of the causes and impacts of climate change is low amongst people under the age of 25 and those over the age of 65 (Bibbings 2004; DEFRA 2003). Despite the mixed perceptions on climate change knowledge and level of awareness reflected in the literature, it is imperative for university students to have knowledge of climate change. This is because it gives them the capacity to devise ways of mitigating its impacts. Wolf and Moser (2011) are of the view that perceptions on climate change issues are thought to contribute to ways of finding possible solutions to the problem. Weber (2010) asserts that through studies such as geography and environmental science, students acquire a more detailed appreciation of climate change than a layman in the community. Apart from fields related to natural sciences and environment, university students are exposed to the scientific and analytical processes that they are anticipated to use in determining the risk and danger of climate change.

Furthermore, in a contrary viewpoint, Brody et al. (2008) and Weber (2010) are of the view that the more educated people (most likely university graduates) are less likely to believe that climate change is risky compared with uneducated people. However, these differences in opinion about climate change issues result in difficulty maintaining commitment to the cause. This is because even though most people report being aware of climate change and its causes, they cannot fully explain its causes, consequences and solutions. Lorenzoni, Nicholson-Cole and Whitmarsh (2007) are of the view that most people believe that climate change is caused by anthropogenic and natural causes although they do not understand the details.

### Climate change as a disaster risk issue

It is widely believed that climate change is beginning to manifest in the form of changing weather and flooding (Kundzewicz et al. 2014). The Global Climate Risk Index (2016) asserts that between 1995 and 2014, more than 525 000 people died worldwide and losses of more than $ 2.97 trillion were incurred as a direct result of over 15 000 extreme weather events. Furthermore, Mugambiwa and Tirivangasi (2017) stress on the fact that climate change will pose a serious threat to the livelihoods of many communities. They argued that:

There are numerous potential effects climate change could have on agriculture. It affects crop growth and quality and livestock health. Farming practices could also be affected as well as animals that could be raised in particular climatic areas. The impact of climate change as well as the susceptibility of poor communities is very immense. (p. 1)

Recently in Southern Africa, the effects of climate change which turned into a disaster risk were felt through tropical cyclone Dineo, which affected some parts of South Africa, Mozambique and Zimbabwe. Consequently, a number of people lost their lives and property. This implies that climate change poses various threats to the environment, economy as well as people’s health. These climate-related risks are ignited by floods, droughts and extremely hot weather conditions. Consequently, it is imperative for university students to have an understanding of the disaster risks posed by climate change as it significantly helps in crafting ways of mitigating its impacts.

#### Objectives

The objectives of this study are as follows:

- To assess the knowledge and understanding of climate change by students at a rural South African university.
- To determine differences in the knowledge and understanding of climate change by students from different faculties.
- To examine differences in the knowledge and understanding of climate change by students based on gender.

#### Research questions

The following research questions were posed:

- Do students at a rural South African university have knowledge and understanding of climate change?
- Are there differences in the knowledge and understanding of climate change by students from different faculties?
- Does the knowledge and understanding of climate change by students differ based on gender?

### Theoretical framework

This study adopted the social constructionism theory. Giddens (2009:160) asserts that, ‘social constructionism is an approach to studying social problems, including environmental problems. Social constructionism suggests that the social is not a given, and so is any knowledge of it’. The assumption is that knowledge of the world is not an objective approach to taken-for-granted knowledge (McHoul & Grace 1993). It has explored how some environmental issues are seen as significant whereas others are seen to be less important or are largely ignored. Social constructionists suggest that all environmental problems are, to some extent, socially constructed by groups of people. Climate change is amongst the most complex challenges that the world is facing. Its impacts considerably contribute to the development challenges of ensuring food security and poverty reduction in the developing world. The phenomenon is broadly viewed as substantial because of its impacts on society, economy and health of the communities. This denotes that university students’ knowledge of climate change is of paramount importance because they significantly contribute to the development of any community in many respects (Amanchukwu, Amadi-Ali & Ololube 2015). The social constructionism provides an important framework for understanding climate change as a serious challenge which students have to understand and for which they are able to devise mitigation measures in future.

## Methodology

The population for the study comprises university students at a rural South African university. The participants were from three faculties of the university, namely, Faculty of Health Sciences, Faculty of Science and Agriculture and Faculty of Humanities. This study utilised a quantitative approach and a descriptive design. Therefore, the findings of the study are based only on how respondents self-report. Convenience sampling was used to obtain respondents. This method was used because participants were easily available. Cooper and Schindler (2008) assert that convenience sampling is a non-probability sampling technique where participants are selected based on ease of access and proximity. Self-administered questionnaires were utilised in a survey to collect data from participants following a similar study by Ojomo et al. (2015). A pilot survey was conducted to pre-test the data collection tool. Calitz (2009) states that a pilot study is an experimental version of the actual research. A pilot study makes it possible to identify and eliminate problems which might be faced later in the study as well as to improve the face and content validity of the questionnaire (Cooper & Schindler 2008). Hazz and Maldaon (2015) are of the view that a sample size of 10% – 20% is appropriate to conduct a pilot study. As such, 15 questionnaires were sent out to university students. This led to the fine-tuning of the questionnaire where some questions were rephrased to suit the English proficiency of participants. Closed-ended questions were used and the questionnaire consisted of two sections: biographical questions and questions relating to climate change dynamics. It was used following similar studies on climate change where the tool showed high levels of reliability and validity. A five-point Likert scale ranging from strongly disagree to strongly agree was used. A sample of 90 university students participated in the survey. Data analysis included descriptive statistics and *T*-tests. Reliability was measured using the Cronbach’s alpha coefficient.

## Discussion

### Response rate and biographical details

A total of 150 questionnaires were distributed to university students of which 90 were returned, providing a response rate of 60%.

Table 1 presents the biographical information of respondents. Majority of the respondents were females (67%), while only 33% were males. The age distribution of the respondents was as follows: 71% were between 21 and 30 years, 28% were 20 years and below, and only 1% were in the age group of 31–40 years. There was no respondent above 40 years. With regard to the level of education, most of the students (93%) were in their undergraduate studies, while 5% were postgraduates and only 2% were enrolled for a diploma or certificate. The questionnaires were sent to three faculties of the university, Faculty of Health Sciences, Faculty of Science and Agriculture and Faculty of Humanities, in order to get a balanced response on climate change. It was discovered that 66% of the respondents came from health sciences, 27% came from science and agriculture and 7% came from humanities.

**TABLE 1:** Biographical details of the participants.

Biographical factors	Percentage
**Gender**	
Male	33
Female	67
**Age**	
20 and below	28
21–30 years	71
31–40 years	1
**Level of education**	
Undergraduate	93
Postgraduate	5
Diploma/certificate	2
**Faculty**	
Health Sciences	67
Science and Agriculture	27
Humanities	7

### Descriptive statistics

Descriptive statistics were utilised to describe the different variables of climate change embedded in the questions. Likert scale questions ranging from (1) strongly disagree to (5) strongly agree were employed.

Table 2 presents descriptive statistics for climate change variables. The results indicate that university students have low knowledge and understanding of climate change (mean = 3.56; s.d. = 0.94). In support of this finding, Mower (2012) asserts that there is less literature on young people’s knowledge and perceptions of climate change. This implies that most of the literature available on climate change knowledge and perceptions is based on general public opinions. Mower (2012) further asserts that many students have a poor understanding of the details of global warming and the causes thereof. This is because in a study by Mower some students were unaware that CO_2_ is a greenhouse gas and a majority of students did not identify water vapour as a natural greenhouse gas. Numerous studies have established that young people often link the ozone hole and general pollution with anthropogenic climate change (Andersson & Wallin 2000; Pekel & Ozay 2005). As a result, it can be established that students’ understanding of climate change is not convincing. Based on the comparison per faculty, it was discovered that students from the Faculty of Science and Agriculture have a better understanding of climate change (mean = 3.28; s.d. = 0.99) compared with students from the Faculty of Health Sciences (mean = 2.12; s.d. = 0.91) and the Faculty of Humanities (mean = 1.12; s.d. = 0.87). The findings are consistent with a similar study by Hargreaves et al. (2003), who posited that people with a formal science qualification have a higher understanding of the process of climate change.

**TABLE 2:** Descriptive statistics for climate change variables.

Statements	*N*	All		Humanities		Health Sciences		Science and Agriculture	
		Mean	s.d.	Mean	s.d.	Mean	s.d.	Mean	s.d.
1. I have thorough understanding of climate change.	90	3.56	0.94	1.11	1.06	2.21	1.12	3.01	1.08
2. The issue of climate change was introduced to me in the class.	90	3.42	1.31	1.06	0.66	2.33	1.08	3.33	0.58
3. I learnt about climate change through the media.	90	3.52	1.18	1.32	0.73	1.45	1.03	3.41	1.09
4. My community is aware of climate change.	90	2.88	0.95	0.60	0.81	1.66	0.88	2.77	1.00
5. My family is aware of climate change.	90	3.46	1.04	0.91	1.21	2.13	0.69	3.45	0.91
6. I am aware of the causes of climate change.	90	2.04	0.89	1.03	0.91	1.49	0.77	2.03	0.88
7. Greenhouse gasses are the major cause of climate change.	90	4.03	1.02	1.44	0.58	2.35	0.56	4.00	0.63
8. Floods are a result of climate change.	90	4.14	1.06	1.32	0.63	2.66	0.72	4.12	1.03
9. Droughts are a result of climate change.	90	4.18	0.99	1.09	0.74	2.33	1.13	4.17	1.06
10. Recently, the effects of climate change have been experienced in my community in the form of floods.	90	2.30	1.29	1.04	0.77	2.15	1.07	2.29	1.12
11. The effects of climate change have been experienced in my community in the form of drought.	90	2.90	1.43	1.66	1.06	2.00	1.12	2.35	0.96
12. Recently, the effects of climate change have been experienced in my community in the form of hot and extremely hot weather.	90	3.93	1.14	1.40	1.11	1.89	0.67	3.66	0.88
13. Climate change has severe effects on agriculture.	90	4.49	0.77	1.07	0.89	2.78	0.88	4.12	1.04
14. Climate change has severe impacts on water supply.	90	4.31	0.86	0.88	0.96	2.98	0.91	3.99	0.77
15. Climate change has severe effects on cultural practices.	90	3.46	1.07	1.12	0.78	1.78	0.96	3.33	0.92
16. Climate change has severe impacts on health.	90	4.32	0.92	1.14	0.79	2.35	0.77	4.25	1.13
17. My community is vulnerable to the impacts of climate change.	90	4.30	1.23	1.00	1.12	2.11	1.13	4.28	1.10
18. In my community, I am participating in climate change mitigation programmes.	90	2.12	1.14	0.88	0.75	1.64	1.09	2.01	1.08
19. My University has programmes on climate change.	90	2.57	0.88	1.10	0.86	2.2	0.89	2.43	0.71
20. The government is playing a leading role in climate change mitigation in my community.	90	2.67	1.25	1.13	0.97	1.88	0.81	2.65	0.86
Scale mean	-	3.43	1.07	1.12	0.87	2.12	0.91	3.28	0.99
Cronbach’s alpha	-	0.83	-	-	-	-	-	-	-

s.d., standard deviation.

Furthermore, on the sources of information about climate change, a significant number of participants (mean = 3.52; s.d. = 1.18) indicated that they learnt more about climate change through the media than in the class (mean = 3.42; s.d. = 1.31), although some participants agreed that they learnt about climate change in the class (mean = 3.42; s.d. = 1.31). However, students should not largely rely on the information they acquire from the media and some seemingly credible textbooks as it is prone to bias. Similarly, a study by Shepardson et al. (2011) found that students’ inaccurate understanding of the greenhouse effect apparently resembled the way it was explained in their textbooks. From another angle, King (2010) discovered numerous errors in earth science textbooks used in the United Kingdom. This explains some of the common misconceptions about climate change issues by students. In essence, it can be established that young people’s scientific understanding of the causes of anthropogenic climate change relies mostly on their formal education.

On issues of climate change awareness, on average, the results indicate that university students as well as their families and communities are not aware of climate change dynamics. However, a study by Cherry (2011) suggested that young people pass the information they gain from their studies on to their parents. This suggests that the information that young people receive about climate change can also benefit a much larger percentage of the population, which includes their families.

This study also discovered that most of the students agreed that greenhouse gasses are the major cause of climate change as indicated by a high mean (4.03). This finding is supported by studies conducted by Lorenzoni et al. (2007) who found that most people report being aware of climate change and its causes and show some concern although they cannot explain in detail its causes, consequences and solutions. In some cases, the public relate climate change with greenhouse effects and climate variability. The lack of understanding on climate change issues affects how people would deal with climate change effects.

Furthermore, on climate change vulnerability, the results indicate that university students strongly feel that their communities are immensely vulnerable to the effects of climate change. This is demonstrated by the number of students who concurred on various vulnerability aspects, namely:

1. The effects of climate change were recently experienced in their communities in the form of hot and extremely hot weather (mean = 3.93; s.d. = 1.14).
2. Climate change has severe effects on agriculture (mean = 4.49; s.d. = 0.77).
3. Climate change has severe impacts on water supply (mean = 4.31; s.d. = 0.86).
4. Climate change has severe impacts on health (mean = 4.32; s.d. = 0.92).
5. My community is vulnerable to the impacts of climate change (mean = 4.30; s.d. = 1.23).

These results suggest that students might not be well informed about what climate change really is, but they have an understanding of the threats it poses to communities. In line with this finding, Weber (2010) asserted that it is reasonable to assume that through studies like geography and environmental science students will have a more detailed appreciation of climate change than the average person. The studies expose them to the scientific and analytical processes that are used to determine the risk and danger of climate change. As a result, an understanding of physical and social vulnerability to climate change regarding the environment impacts the livelihoods and disease will be inculcated in them.

Moreover, the study discovered that university students are not actively participating in programmes aimed at dealing with climate change. The Cronbach’s alpha of 0.83 indicates high levels of reliability of the measures. In relation to this finding, studies by Semenza et al. (2008) and Lorenzoni et al. (2007) found that there is an attitude–behaviour gap in environmental problems. They further asserted that even those who claim to be concerned usually provide a plethora of reasons why they usually do not change their behaviours. Studies also suggest that those who report making changes may not have high behavioural engagement. Changes such as reducing energy use are commonly made for other reasons apart from climate change adaptation or mitigation reasons. Therefore, this could explain the reasons why university students are not involved in climate change programmes.

### *T*-tests

*T*-test is a statistical test used in research to analyse a set of two variables. According to Kim (2015):

*t*-test is usually used in cases where the experimental subjects are divided into two independent groups, with one group treated with A and the other group treated with B. (p. 14)

In this study, this data analysis tool was used to compare mean differences between gender (male and female) amongst university students.

Table 3 shows gender differences in the knowledge and understanding of climate change by university students. As indicated, males have slightly better understanding of climate change. However, the results are not statistically significant.

**TABLE 3:** Gender differences in the knowledge and understanding of climate change.

Male (mean)	Female (mean)	*t*-statistic	Significance level
2.38	2.35	1.66	0.13

Significance > 0.05.

### Recommendations

Based on the findings of this study, the researchers established two recommendations:

- Universities should incorporate climate change topics into the curriculum not only in the science and agriculture modules but also in all faculties. This will enable students from across the university spectrum to grasp the basic knowledge of climate change issues so as to educate their communities on a wide range of these issues.
- There should be programmes to prioritise climate change knowledge, awareness and mitigation where students are encouraged to take an active role. These programmes should include awareness workshops where experts in the field are regularly invited to address students.

## Conclusion

Climate change and its impacts have grave consequences on communities and people’s livelihoods. This research suggests that it is worrying to note that students in this particular South African university have little understanding of climate change issues. It emerged that students are not actively involved in climate change initiatives. It was also discovered that universities, as demonstrated by students’ responses in this study, are doing little to incorporate students into climate change awareness programmes. However, a majority of the students of this study have proven to be aware of the threats that climate change poses to communities. This is imperative as the knowledge and awareness of climate change by university students equip them with skills to deal with its future impacts. This was conducted in line with the social constructionism theory which explores how some environmental issues are seen as significant while others are seen to be less important or are largely ignored. In this case, as climate change is amongst the most complex challenges that the world is facing, its impacts considerably contribute to the development challenges of ensuring food security and poverty reduction in the developing world. Hence, if students were knowledgeable and become involved through being taught about climate change, they could potentially make a great impact.
